# Observations upon the Employment of Compression in Aneurism, with Some Statistical Details

**Published:** 1845-10

**Authors:** 


### Art. XIII.

*Observations upon the Employment of Compression in Aneurism, with some Statistical Details.* By O'B. Bellingham, m.d. f.r.c.s.i., Surgeon to St. Vincent's Hospital.—*Dublin,* 1845. 8vo, pp. 16.

The frequency of the occurrence of aneurism, the extreme difficulty of many of the operations necessary for its cure—testing as they do more closely than any others in the whole range of surgery the skill and knowledge of the operator—and the great uncertainty of their result, even when performed under the most favorable circumstances—render any modification of the established treatment of this disease a subject of peculiar interest and importance to the surgeon. This is more particularly the case if the advocates of the new process hold out the hope that by its adoption the dangers that are attendant on the means ordinarily pursued may, in a great measure, if not wholly, be avoided.

Indebted as we are to John Hunter for many of the most brilliant discoveries in the science, and of the most useful improvements in the practice of surgery, there is none for which we are under deeper obligations, than the knowledge of the true principles that should guide the surgeon in applying a ligature to an artery in a case of aneurism. Shortly after the introduction of the Hunterian operation and the general recognition of its principles by surgeons, it was attempted to be modified by the employment of such pressure to the artery leading to the sac as would obliterate its canal; the pressure being applied to the vessel at a point where its coats were healthy, and not to the tumour itself or its immediate vicinity, as had previously been done by Guattani and others. This plan of treatment, which was pretty extensively tried, was far from successful, and in those cases that did well under it, success was dearly purchased by the extreme pain and irritation to which the patient was subjected.

To secure the obliteration of the artery, various plans were proposed by different surgeons. The following was recommended by Mr. Freer: a moderately tight bandage is first to be applied to the whole limb; a pad is next to be placed over the artery a few inches above the tumour, and then a tourniquet round the limb, the screw of which is to be fixed upon the pad. Care must previously be taken to secure the limb from the action of the instrument by a piece of board wider than the limb itself, by which means the artery only will be compressed when the screw is tightened. The tourniquet should now be screwed up till all pulsation in the tumour ceases; in a few hours the limb will become œdematous and swollen, when the tourniquet may be removed, the pressure of the pad and bandage sufficing for the remainder of the treatment. This process was found by Mr. Freer to succeed in obliterating the radial artery of horses, but was not very successful when applied to the human subject. The instrument used by Sir A. Cooper and Sir W. Blizard, for the purpose of compressing the artery, resembles in its principle the one usually employed at present. "The points of support for this instrument were the outer part of the knee and the great trochanter, a piece of steel passing from one to the other; and to the middle of this a semi-circular piece of iron was fixed, which projected over the femoral artery, having a pad at its end moved by a screw, by turning which, the artery was readily compressed, and the pulsation in the aneurism stopped, without any interruption to the circulation in the smaller vessels." * The patient, however, was unable to bear the pressure for more than nine hours, so great was the agony produced.

Mr. A. White likewise tried pressure in one case with a peculiarly constructed spring. "The woman bore the pain heroically for five days; but the parts compressed sloughed deeply. The cure was completed; but the pain, danger, and risk incurred were infinitely greater than any which could have been sustained from the usual operations."† Lisfranc‡ states that he has seen two cases of popliteal aneurism cured by compressing the femoral artery at the lower third of the thigh, and many in which the employment of this means was unsuccessful. According to Lisfranc compression has been tried by the following surgeons, besides those whose names have just been mentioned: Viricel, Boyer, Collier, Travers, Pelletan, Gualtier, Clement, Dubois, Lyford, and Ehrmann. On collecting all the cases he finds that the successful were to the unsuccessful in the proportion of five to thirteen.

From the foregoing details it would appear that the success attending the treatment of aneurism by compression of the artery leading to the tumour was but trifling, and certainly not sufficient to counterbalance the pain and danger consequent on this method. The cause of this want of success was not so much owing to the compression itself as to the erroneous principles on which it was conducted; the object aimed at by the surgeon in all these cases being to effect a cure in the same manner as when the vessel is ligatured; namely, by its obliteration at the point compressed. In order to accomplish this it was of course necessary to keep up very powerful and long-continued pressure on one spot, the inevitable result of which was the occurrence of insupportable pain, of ulceration and sloughing of the parts compressed, and of much constitutional disturbance. Indeed the local mischief and general irritation occasioned by this plan were such that it was quickly abandoned for the more certain and less painful operation by the knife.

The employment of pressure in cases of aneurism having thus very properly fallen into disrepute, it appears to have been entirely forgotten by surgeons until its revival three years ago by Dr. Hutton, whose attention seems to have been directed to it in consequence of its being unadvisable for several reasons to operate, in the usual way, on a patient of his labouring under popliteal aneurism. From the time of its revival in November 1842 until

* Medical and Physical Journal, vol. viii, p. 2. † Guthrie on Diseases of Arteries, p. 142.

‡ Sur l'Oblitération des Artères, &c. p. 40.

February 1845 it has been had recourse to in twelve cases of femoral and popliteal aneurism, and in all with the most perfect success; for not only has the disease been in every instance speedily cured with comparatively little pain or irritation, but in not one case has it, as yet, we believe, shown a disposition to return. And if the employment of compression in aneurism, as at present conducted, continues to be supported by an amount of success at all approaching that which has hitherto attended it, it cannot but be looked upon as one of the happiest of the triumphs of modern surgery.

The great success that has attended the revival of the employment of pressure to the artery at the site of Hunterian deligation for the cure of aneurism, is no doubt owing to its mode of action being properly understood, and to it being now recognized that the degree of pressure necessary for ensuring the cure of the disease is very much less than what is requisite for the obliteration of the canal of the artery by plastic deposit at the point compressed—the object formerly aimed at, and the accomplishment of which entailed so much suffering upon the patient. On inquiring closely into the particulars of the cases of aneurism that have lately been treated with pressure, it will be found that the femoral artery could be traced, after the cure was accomplished, nearly to the sac of the aneurism, considerably below the point compressed, proving incontestably that the vessel is not obliterated at the site of the pressure, but that the disease is cured in some other way than by the obstruction of the artery leading to it.

In what way then, it will be asked, is the cure effected? In answer to this question we cannot do better than to give Dr. Bellingham's remarks in his own words:

"Upon a former occasion (Dublin Journal, vol. xxiii,) I endeavoured to show that such an amount of pressure as would obliterate the artery is never necessary; and that a cure would be more certainly and more quickly brought about by allowing a feeble current to pass through the sac of the aneurism, than by completely checking the circulation in the vessel. As this principle appears to have been established by the results of the cases which have occurred in this country since, I shall now merely quote what I then said upon the subject.

"When it was considered absolutely necessary for the success of compression, that such an amount of pressure should be applied, as was almost certain to occasion sloughing of the part, and very certain to occasion intense pain and suffering to the patient; and when, in addition, this was to be prolonged through five successive nights and days, we can readily understand why patients refused to submit to it, and we can easily account for the disrepute in which the practice fell, and for the unwillingness of surgeons to adopt this treatment in preference to the simple operation of placing a ligature upon the femoral artery. It would appear, however, that it is not at all essential the circulation through the vessel leading to the aneurism should be completely checked, but rather the contrary; it may perhaps be advantageous at first for a short period; by which the collateral circulation will be more certainly established. But the result of this case, if it does no more, establishes the fact, that a partial current through an aneurismal sac, will lead to the deposition of fibrine in its interior, and cause it, within a few hours, to be filled and obstructed, so as no longer to permit of the passage of blood through it. Pressure so as altogether to obstruct the circulation in an artery must necessarily be slower in curing an aneurism, as it must in some measure act by causing obliteration of the vessel at the part to which the pressure has been applied; whereas a partial current through the sac enables the fibrine to be readily entangled in the parietes of the sac in the first instance, and this goes on increasing, until it becomes filled; the collateral branches having been previously enlarged, the circulation is readily carried on through them." (pp. 5 and 6.)

The object then to be accomplished by the compression of the artery, as at present adopted, is the coagulation of the blood, and the formation of a fibrinous deposit in the aneurismal sac. The flow of blood through the tumour being very greatly lessened, the fibrine is more readily entangled by its lining membrane, and successive depositions of lymph taking place, the sac is completely filled, the tumour becomes solid, and all pulsation in it ceases. As no blood can any longer pass through the sac, the circulation is entirely carried on by the collateral vessels, which have gradually been enlarging proportionately to the obstruction in the sac, and the condition of the aneurismal tumour becomes precisely that of one that is undergoing spontaneous cure.*

Since the revival of the treatment of aneurism by compression of the artery at the Hunterian site, very considerable improvements have been made in the kind of instrument to be used, and in the mode of applying the pressure. The instrument that Dr. Bellingham employs is a modification of a carpenter's clamp, and was invented by a patient of Dr. Harrison's whilst under treatment for popliteal aneurism. It consists of an arc of steel, covered with leather, at one extremity of which is an oblong padded splint, the other extremity terminating in a nut containing a quick screw, to which a pad similar to that of the tourniquet is attached. The principle of this instrument is stated by Dr. Bellingham to be so simple, that the patient can regulate its application himself; and it can be made of every size, so as to compress any vessel within the reach of compression.

The instrument used by Mr. Liston and others is similar in principle though differing somewhat in construction, being simply a horse-shoe tourniquet with well-stuffed pads. It is of considerable importance in practice that the pads be sufficiently broad and square; if too conical they are apt to occasion inconvenient and unnecessary excoriation, or even sloughing, and to allow the vessel to roll away from under them. Mr. Liston has found much advantage in protecting the integuments covering the artery with a piece of soap-plaster, spread on leather, and the outer part of the thigh with a splint of strong sole leather, for the external pad of the instrument to rest against; thus removing all direct pressure from the soft parts, and diffusing it over a greater extent of surface.

The mode of application of the compressor has lately been modified in a very important manner by Dr. Bellingham. This gentleman employs two or three of the instruments at the same time, which are placed upon the artery leading to the aneurism, only one at a time, however, being tightened. When the pressure of this becomes painful it is slackened, one of the others having previously been screwed tight. In this way the pressure may be alternated and continued uninterruptedly for any length of time, the patient being able to make the change in the tourniquets as he

* It has occurred to us that the process of cure of an aneurism by means of compression might be much hastened and rendered more certain even than it at present appears to be, by employing in conjunction with it, the plan recommended some years since by Mr. Phillips, of passing a galvanic current through the tumour, and thus coagulating the fibrine in it. We merely throw out this suggestion for the consideration of those surgeons who may be about to treat any cases of aneurism by the pressure of the artery leading to the tumour.

finds most convenient to himself. Another advantage of this mode of application of the instrument is that the cure will be much expedited in consequence of no time being lost by the parts subjected to pressure becoming excoriated or ulcerating, from the pressure of the pad of any one of the instruments being too long continued.

Conceding, however, the practicability of curing certain aneurisms by the compression of the arterial trunks leading to them, the question naturally arises, Does this plan of treatment possess any, and if so, what advantages over the established practice by ligature? To this important question we are, perhaps, scarcely in a condition to give an answer at present. The number of cases treated by compression is not as yet sufficiently large to warrant us in drawing any general inferences; and although Dr. Bellingham adduces twelve cases of femoral or popliteal aneurism (the only ones in which it has as yet been tried) that have been cured by it, yet this number cannot be considered large enough for the purpose of comparison with the result of the treatment by ligature, for we know surgeons who have tied the femoral artery more than twelve times in succession without losing a patient, or having any disagreeable after-consequences produced.

With this caution to our readers we will enumerate the principal advantages that Dr. Bellingham considers compression to possess over the ligature of an artery in cases of aneurism.

1. In the first place it is not attended by the slightest risk to the patient: whereas, even the most carefully performed operation with the knife is attended by very considerable danger, arising, as Dr. Bellingham very justly remarks, not so much from the disease itself as from the means adopted for its relief.

2. There are certain cases of aneurism in which it would not be safe to ligature the artery, as such a step would probably be followed by unfavorable results. The cases alluded to are those in which the aneurism has been allowed to attain so large a size as to interfere materially with the collateral circulation, compressing the veins in its neighbourhood, producing congestion of the extremity and consequent œdematous infiltration of the tissues. If the vessel be ligatured in these cases it is scarcely possible to prevent the occurrence of gangrene, the collateral circulation not being able to establish itself with sufficient rapidity to preserve the vitality of the limb; and indeed the only resource in a case of the kind alluded to is the removal of the diseased limb. In the treatment of aneurism by compression, on the other hand, the change effected is slow and gradual—the supply of blood is not entirely cut off from the affected part, and can at any moment be restored. Indeed, Dr. Bellingham thinks, and in this opinion we entirely coincide, that pressure is more likely to succeed in curing a large than a small aneurism, inasmuch as the lining of a large aneurismal tumour is not so smooth as that of a small one, and will consequently more readily entangle the fibrine of the blood that flows through it. In support of this opinion we may adduce the cases that have already occurred, in most of which the aneurisms were of considerable size.

3. Again, it is not uncommon for a thinning of the walls of the sac to take place, and for inflammation and suppuration to follow the application of the ligature. This has not as yet occurred after the use of compression, although in several of the cases in which it was had recourse to the tumour was of very large size, and in Mr. Cusack's case the parietes were so much thinned, that great fears were entertained that they would give way.

4. Another case in which the application of a ligature is attended with much danger, is when there is such disease of the coats of the artery that the thread cuts its way through them without exciting the necessary adhesive inflammation. In this case compression would be as likely to be followed by favorable results as in any others, the object being not to obliterate the vessel at the point compressed, but merely to retard the passage of the blood through the sac.

5. There are other less common cases, those of the aneurismal diathesis for instance, or when the patient has an unconquerable dread of the knife, in which the application of a ligature to an artery is inadmissible, but in which compression may very safely be had recourse to.

6. Lastly, should pressure fail in curing the aneurism, which Dr. Bellingham thinks very unlikely from the results hitherto obtained, its employment would not preclude the subsequent operation in the ordinary way; " but by retarding the increase of the aneurism, and assisting in the establishment of a collateral circulation, it would tend rather to render the chances of the operation by ligature more favorable."

Such being the advantages that compression possesses over the ligature in the treatment of aneurism, what, it will be asked, are the objections to its employment?

The first that has been urged is that the arteries to which this plan of treatment is really applicable, are but few in number. But Dr. Bellingham has a sufficient answer to this:

" What is really the fact? The artery above all others in which aneurism is most frequent after the aorta, is the popliteal, and next in frequency come the femoral and the brachial. Lisfranc has given a table of 179 cases of aneurism, (exclusive of those of the aorta,) collected from various works, and of this number the popliteal artery was engaged in 59 instances, while the carotid was engaged 17 times, the subclavian 16, and the external iliac only 5 times. But even this must be much below the average, for few cases, comparatively, of operations for popliteal aneurism have been published, owing to its frequency, unless there happened to have been some peculiarity in the case; whereas most of the operations upon the iliac, subclavian, and carotid arteries, have been brought before the profession on account of the unfrequency of disease in those vessels. It must be recollected also that aneurism of the subclavian, or carotid arteries near their origin, and of the common iliac or inominata, which do not admit of the application of compression do not admit either of the employment of the ligature. It surely therefore ought not be urged against this method, that because aneurism occurs in arteries beyond its reach, we should refuse to apply it to vessels to which it is adapted; or that the practice should be denounced because it is not applicable to every vessel." (pp. 9-10.)

Another objection that has been made to the plan of treatment under consideration, is the likelihood of the return of the pulsation in the tumour. It is difficult at present to answer this objection in a way that will be satisfactory to all, as it may be urged that the cases treated by compression have not been treated for a sufficient length of time to make it certain whether the disease has been effectually cured or not; for it is well known to every surgeon that after the ordinary operation an aneurism may return at a very late period. Thus, Sir A. Cooper has related a case in which fifteen years elapsed before the tumour returned; and Mr. Cæsar Hawkins one in which no pulsation appeared for seven years after the artery had been ligatured, but then it did return, and the limb was amputated. These, however, may justly be considered exceptional cases; and we think that if the pulsation has not returned in a twelvemonth after the operation, we may very fairly consider the disease to be cured. Now in several of the instances referred to in Dr. Bellingham's paper, in which compression has been had recourse to, two and in some nearly three years have elapsed, and in few less than a twelvemonth; yet in none has the disease to our knowledge reappeared. And indeed there is no reason for supposing that the pulsation would be more likely to return after the cure by compression than after that by ligature of the vessel. On the contrary, from the manner in which the cure is effected when compression is employed, the sac being gradually filled up by a mass of solid fibrine and not, as when the ligature is had recourse to, by a loosely-formed coagulum, the disease would appear to us to be less likely to return after its cure by compression, than by the ligature.

The last objection worth noticing is that this mode of treating aneurism is more tedious and painful than the ligature of the artery. Several of the cases published in which the pulsation ceased in the tumour after compression had been employed for a few days only, would tend to show that it is not necessarily more tedious than the treatment by tying the vessel. And even supposing it to occupy double the time required for the cure of an aneurism by the successful application of the ligature, if it can be shown to be less likely to be attended with dangerous consequences, and to afford a greater chance of safety to the patient, few surgeons would be rash enough to run an unnecessary risk in order to save a few additional days of a very simple treatment.

There is as little foundation for the assertion that the treatment by compression is more painful than that by the ligature from the first incision with the knife to the removal of the last dressing. As formerly employed, when the object was to obliterate the artery at the point compressed, it no doubt was most painful and tedious. But as now practised, as we have had occasion to observe in two instances of its employment, more particularly if the very important improvement of having two or three compressors on the limb, as suggested by Dr. Bellingham, is had recourse to, the treatment will be found to occasion but very trifling uneasiness to the patient.

The following are the principal deductions that Dr. Bellingham draws from the preceding inquiry:

"1st. That the arteries to which pressure is applicable, being far more frequently the subject of spontaneous aneurism than those to which it is inapplicable, compression promises to supersede the ligature in the great majority of cases.

"2d. Pressure has several obvious advantages over the ligature, being applicable to a considerable number of cases in which the ligature is contra-indicated or inadmissible.

"3d. The treatment of aneurism by compression does not involve the slightest risk; and even if it should fail, its employment not only does not preclude the subsequent operation by ligature, but renders the chances of the operation by ligature more favorable.

"4th. Such an amount of pressure is never necessary as will cause inflammation and adhesion of the opposed surfaces of the vessel at the point compressed.

"5th. Compression should not be carried even so far as completely to intercept the circulation at the point compressed; the consolidation of the aneurism will be more certainly and more quickly brought about, and with less inconvenience to the patient, by allowing a feeble current of blood to pass through the sac of the aneurism.

"6th. Compression by means of two or more instruments, one of which is alternately relaxed, is much more effectual than by any single instrument.

"7th. Compression according to the rules laid down here is neither very tedious nor very painful, and can be maintained in a great measure by the patient himself.

"8th. An aneurism cured by compression of the artery above the tumour, according to this method, is much less likely to return than where the ligature had been employed." (pp. 13, 14.)

The employment of compression to the artery above the aneurismal tumour, according to the principles on which it is at present adopted, is as yet but in its infancy, and time alone will enable us to form a proper estimate of its value. The results, however, that have already been obtained by it are of the most satisfactory character, and such as must induce every surgeon to hesitate before he again applies a ligature, in a case of aneurism, to an artery within the reach of compression. To all those who wish to obtain full information of the present state of this very important subject, we would strongly recommend the perusal of Dr. Bellingham's very valuable pamphlet, which contains in a few pages everything of importance connected with it.

---

